# Incorporation and deposition behaviors of Zn into the channel of hydroxyapatite

**DOI:** 10.1039/d5ra06364g

**Published:** 2025-10-14

**Authors:** Xiao Chen, Kanji Saito, Takashi Toyao, Yuriko Ando, Ken-ichi Shimizu, Masataka Ogasawara, Sumio Kato

**Affiliations:** a Department of Materials Science, Graduate School of Engineering Science, Akita University 1-1 Tegatagakuen-machi, Akita-shi Akita 010-8502 Japan saitok@gipc.akita-u.ac.jp katos@gipc.akita-u.ac.jp; b Kagami Memorial Research Institute for Materials Science and Technology, Waseda University 2-8-26 Nishiwaseda, Shinjuku-ku Tokyo 169-0054 Japan; c Institute for Catalysis, Hokkaido University N-21, W-10 Sapporo 001-0021 Japan

## Abstract

Zn was successfully incorporated into the channel of hydroxyapatite by loading of Zn and subsequent heat treatment, leading to a Hap with the largest Zn content in the channel compared with those obtained by the conventional wet chemical process. The incorporation of Zn into the channel was supported by X-ray diffraction analysis combined with Rietveld refinement, infrared spectroscopy, Raman spectroscopy and X-ray absorption spectroscopy analysis. Moreover, we demonstrated that the incorporated Zn was deposited from the channel in the form of ZnO fine particles. Homogeneous distribution of ZnO on hydroxyapatite particles was confirmed by elemental mapping using transmission electron microscopy, suggesting the potential of this methodology to serve as a way of addressing metal oxide-decorated hydroxyapatite, which is attracting attention in the field of catalysis.

## Abstract 2

Incorporation/release of Zn into/from the hydroxyapatite (Hap) channel upon heat treatment under varying humidity were investigated to find the Hap with the largest Zn content in the channel so far as well as deposition of ZnO fine particle.

## Graphical abstract

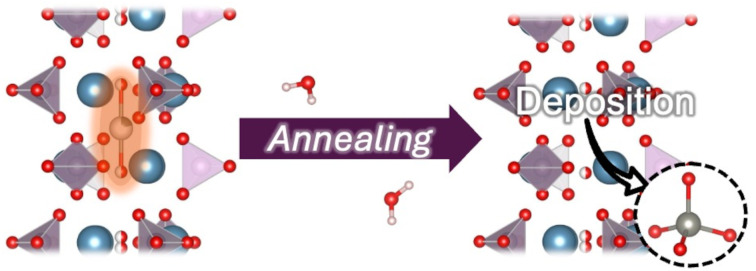

## 1 Introduction

Incorporation/release of metal elements into/from host inorganic crystals is a key objective, not only as a means of concentrating (removing) rare/toxic elements from the environment, but also from the perspectives of addressing unique compositions and structures. Cation/anion incorporation into nanospaces provided by inorganic exchangers (*e.g.*, zeolites, clays, phosphates, transition metal oxides, hydroxides) through a solution route^1–6^ or solid-state reaction^7–10^ at or near room temperature has been investigated. There have also been cases of ion-exchange reactions being driven under high temperatures.^11,12^ As well as ion-exchange reactions, reversible incorporation/deposition behaviors of metal cations into/from host lattices depending on the atmosphere under high temperature have been investigated. A kind of perovskite-type compound demonstrated that such novel metals as Pt, Rh and Pd were passed back and forth between a perovskite framework (bulk) and the surface in response to partial pressure of oxygen upon heat treatment, suggesting the usefulness of this phenomenon in the design of self-generative catalysts (support catalysts often suffer damage from sintering).^13,14^ The further potential of materials functionalization driven by elemental exchange across solid interfaces is an intriguing topic.

Apatite-type compounds with the general formula A_10_(MO_4_)_6_X_2_, which comprise A cations (A = Ca, Sr, Ba, *etc.*), MO_4_ tetrahedra (M = P, Si, Cr, Mn, V, As, *etc.*) and X anions (*e.g.*, OH^−^, F^−^, Cl^−^), have been investigated in a variety of fields, including medicine, energy, the environment and electronics/optics, depending on their composition. Hydroxyapatite (Ca_10_(PO_4_)_6_(OH)_2_; Hap), which is an apatite-type compound found in bones and teeth, has been of great interest for varied applications such as biomaterials^15–18^ and catalysts^19–22^ that benefit from its bio-compatibility and thermal resistance, combined with its abundance (low-cost). Structural elements (*e.g.*, calcium and phosphorous) have often been substituted with appropriate heteroelements to enhance/expand their potential as materials according to the intended purpose. In addition to isomorphous substitution, incorporation of metal cations into the interstitial site, which is located in a channel surrounded by A cations running parallel to the *c*-axis of the apatite structure,^23–33^ has attracted increasing attention as a way of modifying the Hap functions. A unique characteristic of Hap whose channel is occupied by metal cations is a red color demonstrated by Cu-containing Hap.^34^ The color varies depending on the A cation species (purple and blue for Sr and Ba, respectively),^35^ encouraging subsequent research into channel modification with/without isomorphous substitution for controlling optical,^36–41^ magnetic,^42–44^ and biological properties.^45^ Hap containing metal in the channel has conventionally been synthesized by solid-state reaction at 400–1100 °C with multiple calcination steps. We have demonstrated, on the other hand, that a guest cation (Cu ion) can be incorporated into the channel by post-treatment, in which the target metal is loaded onto Hap powder by the impregnation method and the resulting mixture is subsequently annealed.^46^ It was suggested that the charge balance was maintained by replacement of OH^−^ with O^2−^ (proton dissociation), as seen in the following eqn (1),^47^ assuming divalent copper:

1 CuO + 2[OH^−^] ⇄ [O^2−^–Cu^2+^–O^2−^] + H_2_O

We found, moreover, that the incorporated Cu ion was deposited as an oxide from the channel upon heat treatment.^46^ As is evident from the above equation, the incorporation/deposition of the Cu ion into the channel was accompanied by water release/uptake, and the amount of Cu incorporated/deposited accordingly depended on water content in the atmosphere.^47,48^ The post-synthetic method of incorporating Cu into the channel was applicable to other types of transition metals (Fe,^49^ Ni^50^ and Mn^51^), earning it credit for controlling the composition of Hap containing metal in the channel. Also using this methodology, we have successfully demonstrated that multiple metals can be incorporated into the channel.^50–52^ With the help of co-incorporated Cu, the deposition of Ni^50^ and Mn^51^ from the channel was facilitated. With Ni deposited as NiO along with CuO, deposition of Cu and Mn resulted in the formation of a spinel-type double oxide (Cu, Mn)_3_O_4_,^51^ whose morphological characteristics (fineness) suggested its possible use as a supporting catalyst. These findings suggest the beneficial potential of the methodology we developed, not only for controlling the composition of Hap containing metal in the channel, but also for designing functional Hap materials decorated by metal–oxide nanoparticles.

The synthesis and characterization of Hap containing Zn, which is known as the most abundant trace impurity in bones, has been investigated from the perspectives of bone formation and the mineralization mechanism,^53^ as well as that of designing implant materials with improved bone formation properties around the material^54–56^ and controlled biochemical characteristics.^57–59^ In the field of materials science, Hap, whose Ca is substituted by Zn, has been of interest as adsorbents,^60^ photocatalysts^61–63^ and catalyst-supports,^64^ in addition to as biomaterials.^65–69^ The effects of sites (the Ca site and channel interstitial site) to be doped have also been examined in terms of the rate of Zn elution correlated to biotoxicity and antibacterial properties.^70^ While Hap whose Ca is replaced by Zn with the desired Zn content has been synthesized benefitting from the development of synthetic methodologies such as the ion-exchange^71,72^ and mechanochemical methods,^73–75^ Hap containing Zn in the channel, which has been conventionally synthesized by the wet chemical process,^30,70,76–80^ often suffers because of the difficulty of inheriting a nominal composition. When the composition of Hap containing Zn in the channel is described as Ca_10_(PO_4_)_6_Zn_*x*_(O,OH)_2_, accordingly, the maximum *x* has been limited to 0.25 (up to 2.2 mol% with respect to Ca). The products, moreover, are often obtained as mixtures with by-products such as β-tricalcium phosphate (β-Ca_3_(PO_4_)_2_, β-TCP). Synthesis of single-phase Hap with increased Zn content into the channel achieved by adopting the post-synthetic methodology we developed is thus of interest. In addition, we examined the deposition behavior of Zn from the channel. Hap decorated with semiconductor nanoparticles such as ZnO and TiO_2_ has been investigated intensively as photocatalysts in expectation of a reactant-concentration effect of Hap support,^81,82^ leading us to examine the electronic as well as the morphological characteristics of the deposited ZnO. To the best of our knowledge, there is no report on examining the characteristics of metal species deposited from the channel, except for our report on deposited (Cu, Mn)_3_O_4_ with a morphological focus.^51^

## 2 Experimental

### 2.1 Materials

Ca(CH_3_COO)_2_·H_2_O (99.0%), KH_2_PO_4_ (99.0%) and KBr were purchased from Wako Pure Chemical Industries, Ltd. HNO_3_ (60.0%), KOH (85.0%), ZnO (>99.0%) and Zn(NO_3_)_2_·6H_2_O (99.0%) were supplied by Nacalai Tesque, Ltd. Methylene blue (>98.5%) was obtained from Kanto Chemical Industry Co., Ltd.

### 2.2 Synthesis of Zn-incorporated hydroxyapatite

Hap was prepared by the previously reported method for single phase Hap formation.^46,47,49–52^ Ca(CH_3_COO)_2_·H_2_O and KH_2_PO_4_ with a molar ratio of 9.8 : 6 was added to distilled water. 4 mol L^−1^ HNO_3_ was added to the suspension to dissolve the solids. The pH of the resulting solution was adjusted to 12.5 with 2 mol L^−1^ KOH solution to obtain a suspension. The precipitate was aged at 60 °C for 24 h and then washed five times with water by filtration. The recovered solids were dried at 60 °C. Hap thus obtained was wetted with an aqueous solution of Zn(NO_3_)_2_·6H_2_O in a molar ratio of Ca : Zn = 10 : *x* (*x* = 0–0.6) and dried at 60 °C for 3 h. The resulting powder was pressed into pellets and heated for 3 h at 1150 °C in air, or at 900 °C in N_2_ at a dewpoint below −50 °C, to examine the effect of water content on the amount of Zn to be incorporated. The Ca_10_(PO_4_)_6_Zn_*x*_O_*y*_H_*z*_ products obtained in air and N_2_ are referred to as Zn_*x*_-Hap (wet) and Zn_*x*_-Hap, respectively.

### 2.3 Decoration of hydroxyapatite with ZnO

To study the deposition behavior of Zn ions from the channel, Zn_0.4_-Hap was annealed at *T* °C (*T* = 600–1000) in air for 3 or 9 h. To form ZnO directly on the surface of Hap for purposes of comparison, the pure Hap powder was wetted with Zn(NO_3_)_2_·6H_2_O solution in a molar ratio of Ca : Zn = 10 : 0.4 and dried in air at 60 °C for 3 h. The powder was then pressed into pellets and heated in air at *T* °C (*T* = 600–1000) for 3 h.

### 2.4 Characterization

Phase determination of the products was performed by X-ray diffraction (XRD) using a Rigaku Ultima IV diffractometer operated at 40 kV and 40 mA with Ni-filtered Cu-Kα radiation. The lattice parameters were estimated using the Pawley method. Zn K-edge X-ray absorption spectra (XAS) were recorded in the transmission mode at the BL01B1 beamline with a Si(111) double crystal monochromator (proposal 2024B1749). The powder samples were pelletized into a 7 mm diameter disk and mounted on a sample holder. For purposes of comparison, the spectra of bulk ZnO and Zn foil were also acquired. All spectra were recorded at room temperature. The XAS results were analyzed using Athena software ver. 0.9.25, which is included in the Demeter package.^83^ Infrared (IR) spectra were recorded by the KBr disk method using a JASCO FT/IR-4700 spectrophotometer. Raman spectra were recorded using a Renishaw inVia reflex spectrometer with a 532 nm laser. Ultraviolet-visible diffuse reflectance (UV-vis DR) spectra were obtained using a JASCO V-750 spectrophotometer equipped with an integrating sphere ISV-9221. Transmission electron microscopy (TEM) images were obtained with a JEOL JEM-2100F transmission electron microscope, using an accelerating voltage of 200 kV.

### 2.5 Photocatalytic dye decomposition

The photocatalytic activity of the products was evaluated by decomposition of methylene blue (MB) dye in water. 200 mg of the sample was dispersed in 25 ml of distilled water by ultrasonication for 5 min in a glass vial. Subsequently, 25 ml of 10 ppm MB solution was added to the dispersion and stirred using a magnetic stirrer in the dark until the adsorption reaction of MB onto the solids reached equilibrium. The dispersion was then irradiated with light using a 150 W Xe lamp as a light source under stirring. 5 ml of the dispersion was removed after each 0.5 h and centrifuged to remove the solids from the suspension. The absorbance of the supernatant at 664 nm was measured using a UV-vis spectrometer (JASCO V-750) to determine *C*/*C*_0_, where *C*_0_ and *C* represent the initial concentration and the residual MB concentration, respectively. The solids remaining in the solution was separated from a supernatant by centrifugation, and then dried at 60 °C for 12 h to be characterized by XRD analysis.

## 3 Results and discussion

### 3.1 Zn incorporation into hydroxyapatite

**Fig. 1:**
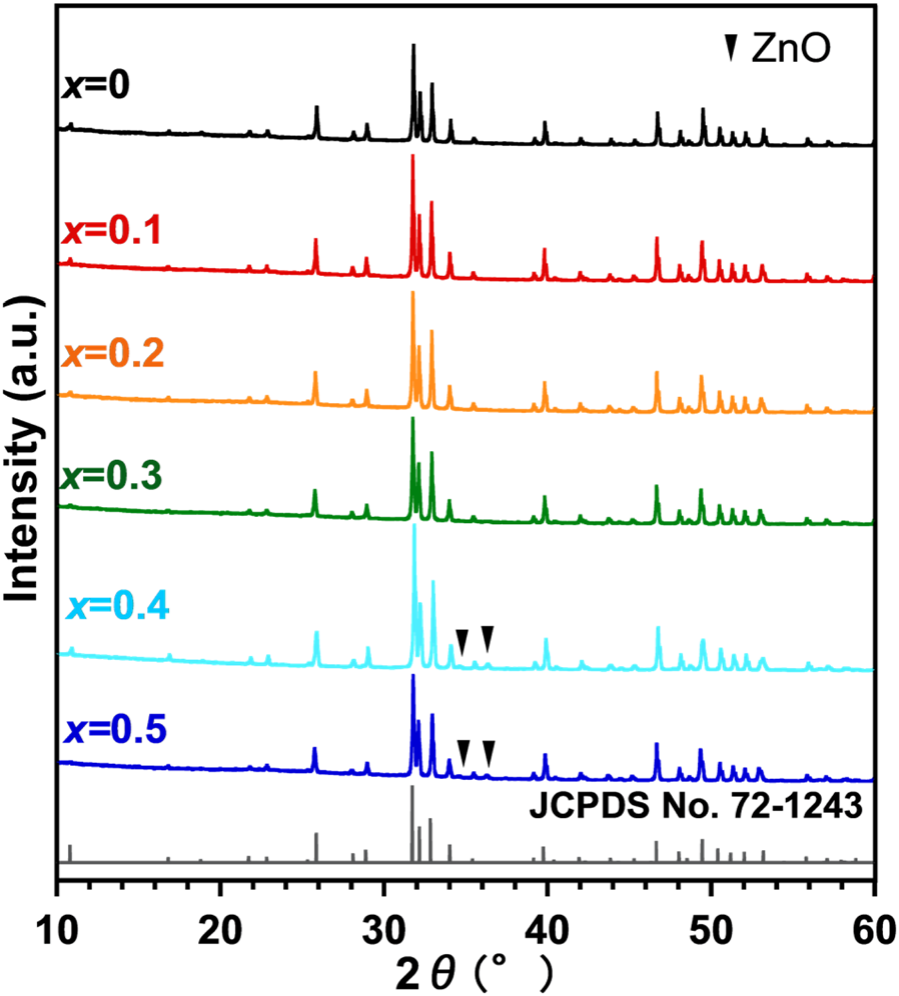
XRD patterns of Zn_*x*_-Hap (wet).

**Fig. 2:**
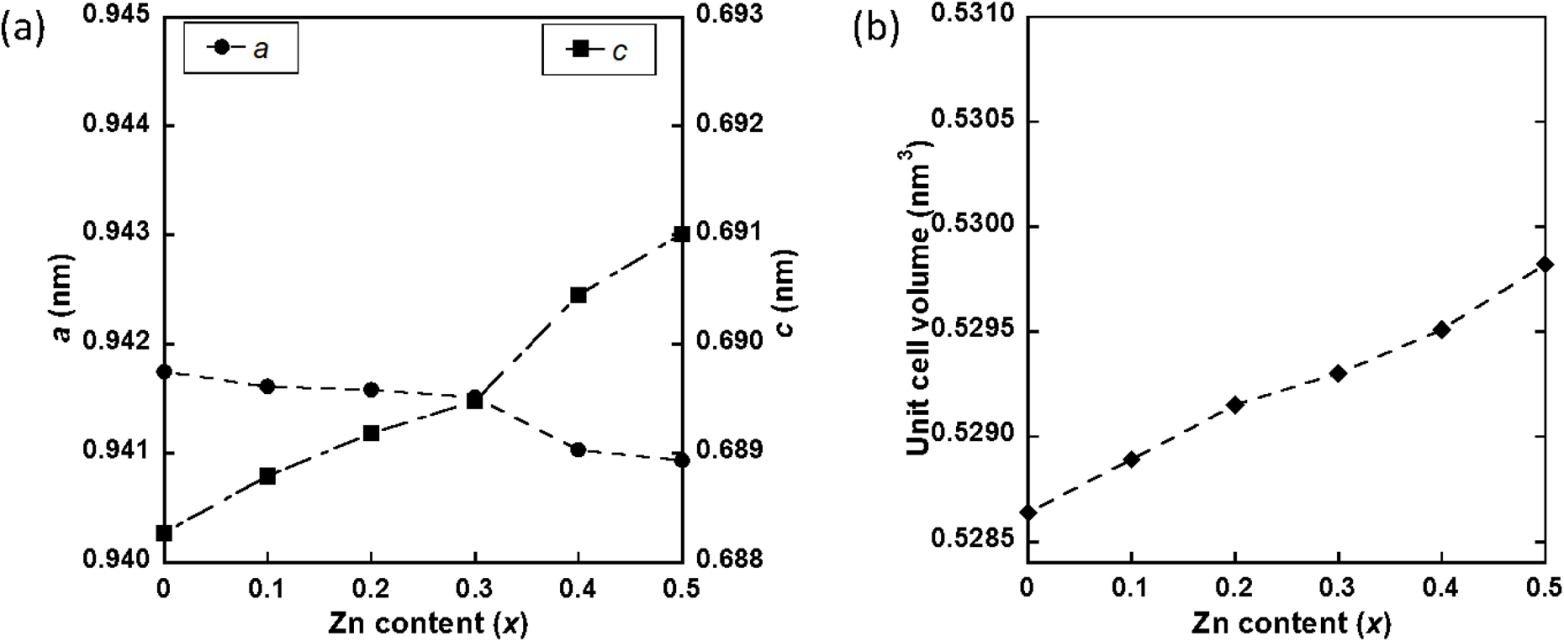
Lattice parameters (a) and unit cell volumes (b) of Zn_*x*_-Hap (wet) as a function of *x*.

The XRD patterns of Zn_*x*_-Hap (wet) are shown in Fig. 1. All the XRD lines were attributable to Hap for *x* = 0–0.3. For *x* ≥ 0.4, on the other hand, diffraction lines attributable to ZnO in addition to those ascribable to Hap were observed at 2*θ* = 34.4 and 36.4°. The lattice parameters and unit cell volumes of Zn_*x*_-Hap (Table S1) as a function of *x* are shown in Fig. 2a and b, respectively. The lattice parameter *a* decreased, while *c* increased with increases in *x*, leading to an increase in the unit cell volume with increases in *x*. Given that the ionic radius of Zn^2+^ (0.74 Å) is smaller than that of Ca^2+^ (1.0 Å)^84^ and that the lattice volumes decreased in the case of isomorphous substitution of Zn by Ca,^67^ the lattice volume increase suggests Zn incorporation into the channel. The correlation between the Zn content and the lattice parameters (*a* and *c*) together with the unit cell volume agree with the previous study on Zn incorporation into the channel of Hap at 1100 °C in air,^77^ further supporting successful Zn incorporation without collapsing the host apatite structure by means of the post-synthetic route.

**Fig. 3:**
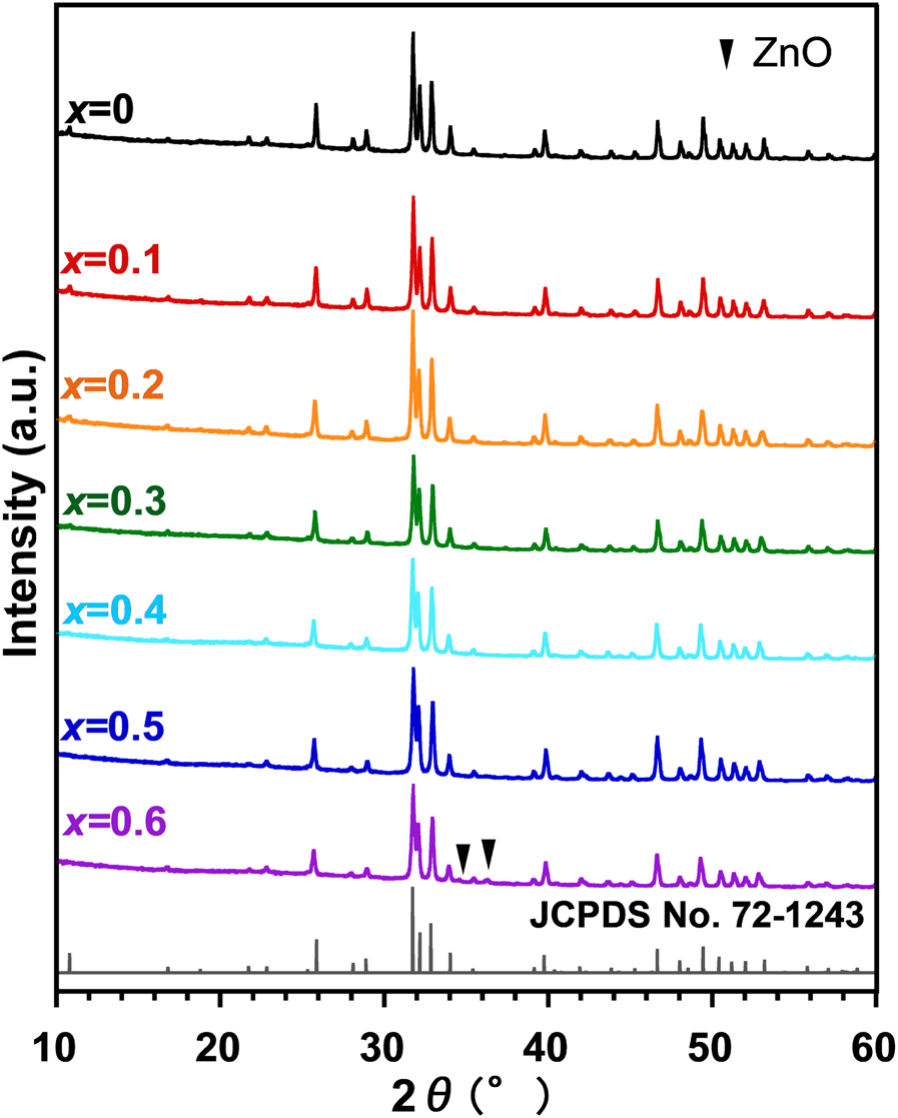
XRD patterns of Zn_*x*_-Hap.

The XRD patterns of Zn_*x*_-Hap, obtained by calcination of Zn-loaded Hap under a dry atmosphere (not in air), are shown in Fig. 3. All the XRD lines were attributable to Hap for *x* = 0–0.5, while the XRD lines ascribable to ZnO were additionally observed for *x* = 0.6. As in the case of calcination in air, larger lattice parameters and a greater unit cell volume were observed with a larger Zn content *x* (Fig. S1). These results suggest formation of single-phase Hap containing Zn in the channel in the case of *x* ≤ 0.5. The larger content of incorporated Zn when compared to that of calcination in air (*x* = 0.3) is ascribable to lower water content in the atmosphere, which promotes Zn incorporation from a thermodynamic (equilibrium) perspective assuming eqn (1). This trend agrees with earlier reports on facilitated metal-incorporation into the channel owing to lower moisture concentrations.^47^ The maximum *x* value of 0.5 (equivalent to 5 mol% with respect to Ca) in this study is remarkably higher than the previously reported value (up to 2.2 mol%),^30,70,76–79^ which was achieved by means of a wet-chemical process, demonstrating the usefulness of the post-synthetic route in increasing the content of Zn incorporated into the channel. The still-low Zn content compared to the theoretical value (*x* = 1.0), which is estimated assuming that the excess positive charge arose from Zn^2+^ incorporation is compensated for proton (H^+^) release,^47,48^ is still under investigation.

**Fig. 4:**
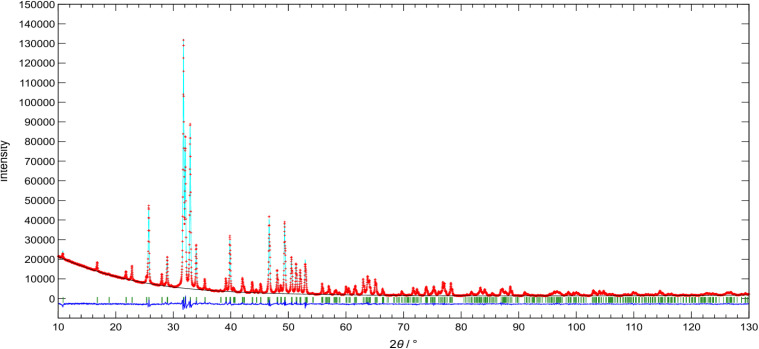
XRD pattern of Zn_0.4_-Hap; experimental (crosses) and calculated (solid line) curves of the Rietveld refinement. The bottom lines show the difference between the two curves. The positions of the apatite phase are shown as vertical lines.

While the trend in the lattice parameters (decrease in *a* and increase in *c* with increasing Zn content) is in good agreement with previous reports on Hap, where Zn occupied the 2b Wyckoff site located at the center of the channel,^76–78,80^ it also agrees with the cases of Fe^49^ and Co,^43,44^ which occupied the 12i Wyckoff site, displaced from the center of the channel. In order to determine the site occupied by Zn in the apatite lattice, Rietveld analysis was performed on Zn_0.4_-Hap. Table 1 shows the refined structural parameters assuming that Zn occupies the 2b site, and the XRD pattern of the refined results is shown in Fig. 4. The Rietveld refinement yielded *R*_wp_ = 3.591% and *R*_F_ = 1.026%, with a Zn content of 0.407, which closely matches the nominal composition. When the structure was refined assuming that Zn occupies the 12i site, however, higher results of *R*_wp_ = 3.729% and *R*_F_ = 1.175% were obtained compared to those for the 2b site (Table S2), suggesting that the opinion that Zn occupied the 2b site was realistic.

**Table 1:** Refined structural parameters of Zn_0.4_-Hap assuming that the Zn occupies the 2b site^a^

Atom	Site	*g*	*n*	*x*	*y*	*z*	U (100 nm^2^)
O1	6h	1	6	0.3284(3)	0.4842(3)	1/4	0.0127(8)
O2	6h	1	6	0.5864(3)	0.4638(3)	1/4	0.0122(8)
O3	12i	1	12	0.3415(2)	0.2560(2)	0.07693(2)	0.0171(6)
O4	4e	0.5	2	0	0	0.2858(14)	0.0191(19)
P	6h	1	6	0.3986(1)	0.3686(1)	1/4	0.0119(3)
Ca1	4f	1	4	1/3	2/3	0.0016(2)	0.0128(3)
Ca2	6h	1	6	0.2462(1)	−0.0075(1)	1/4	0.0139(2)
Zn	2b	0.203(3)	0.407(5)	0	0	0	0.038(2)

a Space group; *P*6_3_/*m* (no. 176); *a* = 0.94075(12) nm, *c* = 0.69113(6) nm, *V* = 0.52971(11) nm^3^, *R*_wp_ = 3.591%, *R*_F_ = 1.026%.

**Fig. 5:**
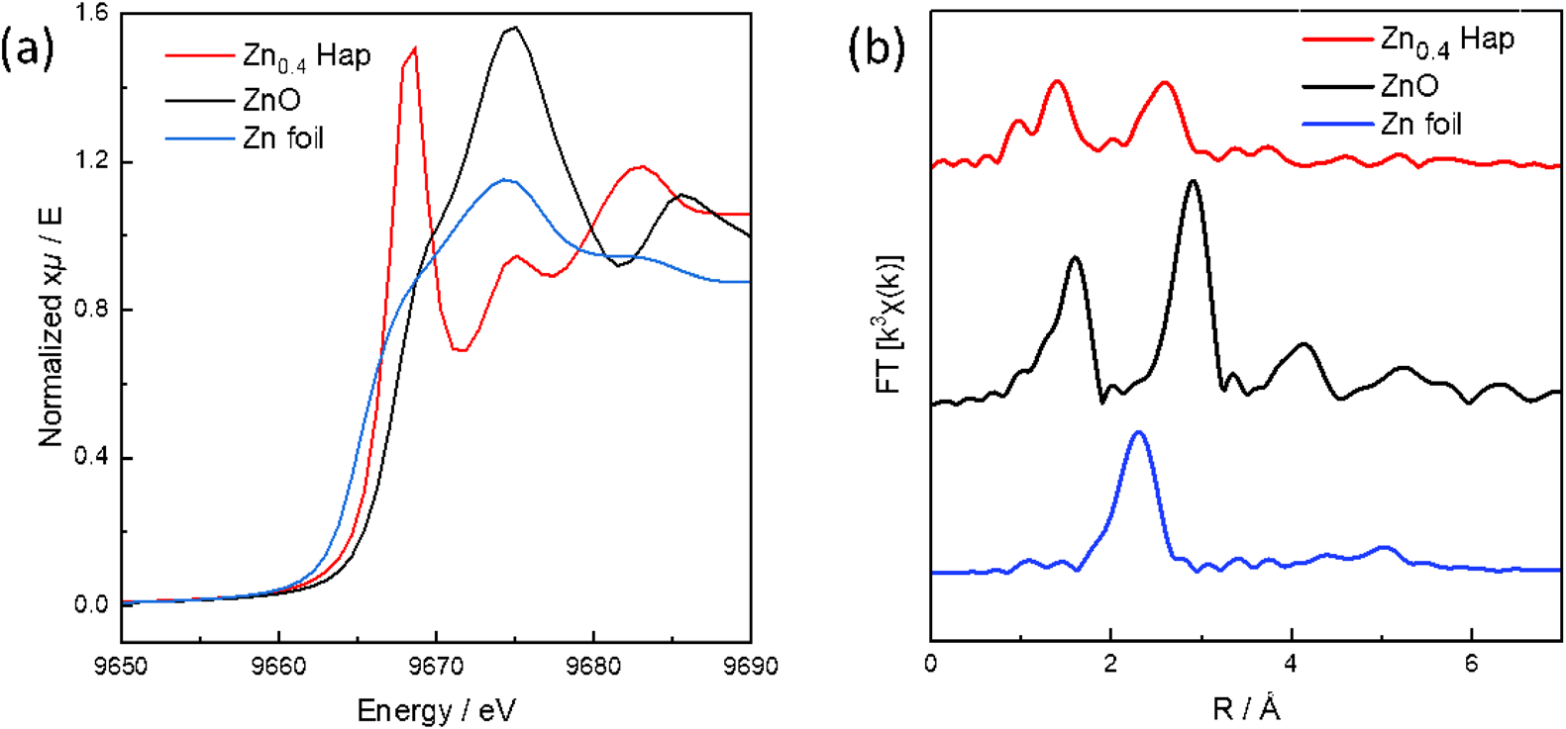
Zn K-edge (a) XANES and (b) Fourier transform of *k*^3^-weighted of EXAFS spectra of Zn_0.4_-Hap and their reference compounds.

Zn K-edge X-ray absorption near-edge structure (XANES) spectrum of Zn_0.4_-Hap and their reference compounds, including ZnO and Zn foil, are shown in Fig. 5. The XANES spectrum of Zn_0.4_-Hap differs from those of both Zn foil and bulk ZnO, with its absorption edge position located between the two, indicating a distinct local electronic structure. Fourier transform (FT) of *k*^3^-weighted extended X-ray absorption fine structure (EXAFS) of Zn_0.4_-Hap show distinct peaks at around 1.4 Å and 2.6 Å (without phase shift correction), which can be assigned to Zn–O and Zn–O–Ca contributions, respectively. Curve-fitting analysis of the EXAFS of Zn_0.4_-Hap, summarized in Table 2, reveals a coordination number (CN) of 1.5 for the Zn–O path at 1.73 Å and a CN of 5 for the Zn–O–Ca path at 2.92 Å, respectively. Given that Zn is coordinated by two O atoms when Zn occupies the 2b site,^30,70,76–80^ the CN for the Zn–O path (1.5) suggests formation of [O–Zn–O] units in the channel of Zn_0.4_-Hap,^78^ while the CN for the Zn–O–Ca path of 5 is in agreement with that estimated based on the refined structure (6).

**Table 2:** Results of curve-fitting analysis of EXAFS spectra of Zn_0.4_-Hap

Shell	Coordination number	Bond distance (Å)	Debye–Waller factor (Å^2^)	Residual factor (%)
Zn–O	1.5	1.73	0.003	4.9
Zn–O–Ca	5.0	2.92	0.010	

**Fig. 6:**
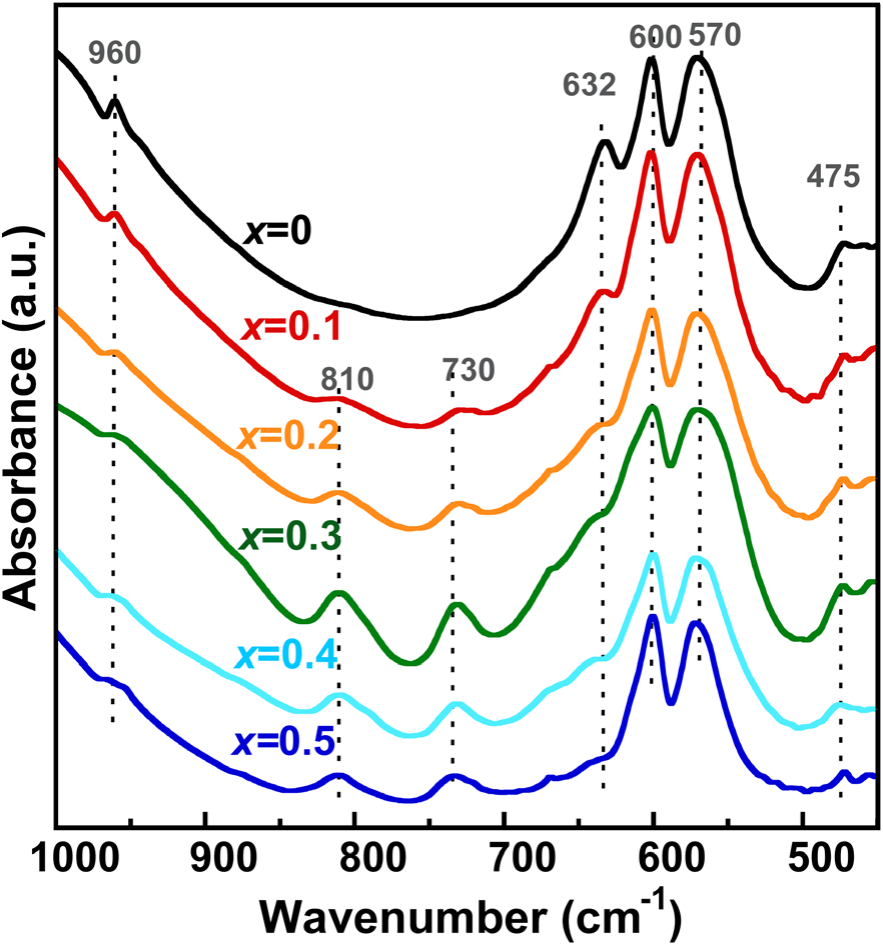
IR spectra of Zn_*x*_-Hap.

To discuss the mechanism of the charge compensation for the excess positive charge arising from Zn incorporation, proton dissociation from OH^−^ into the channel was examined using IR spectroscopy. The IR spectra of Zn_*x*_-Hap are shown in Fig. 6. In the case of pure Hap (*x* = 0), absorption bands attributable to the vibrational modes of PO_4_^3−^ were observed at 475, 570, 600 and 960 cm^−1^, while a band assignable to the stretching vibration of OH^−^, which is located in the channel, was observed at 632 cm^−1^.^85,86^ The intensity of the band attributable to OH^−^ decreased with increases in the Zn content, indicating a decrease in OH^−^ accompanied by Zn incorporation. This suggests that the excess positive charge arising from Zn ion incorporation was maintained by the conversion of OH^−^ to O^2−^, which is in agreement with the case of Cu^2+^ incorporation according to eqn (1). In addition, the intensity of the bands at 730 and 810 cm^−1^ increased with increases in the Zn content for *x* = 0.1–0.5. The appearance of these bands is likely correlated to the afore-mentioned [O–Zn–O] units formed in the channel, whose presence is confirmed by the XAFS measurements.

**Fig. 7:**
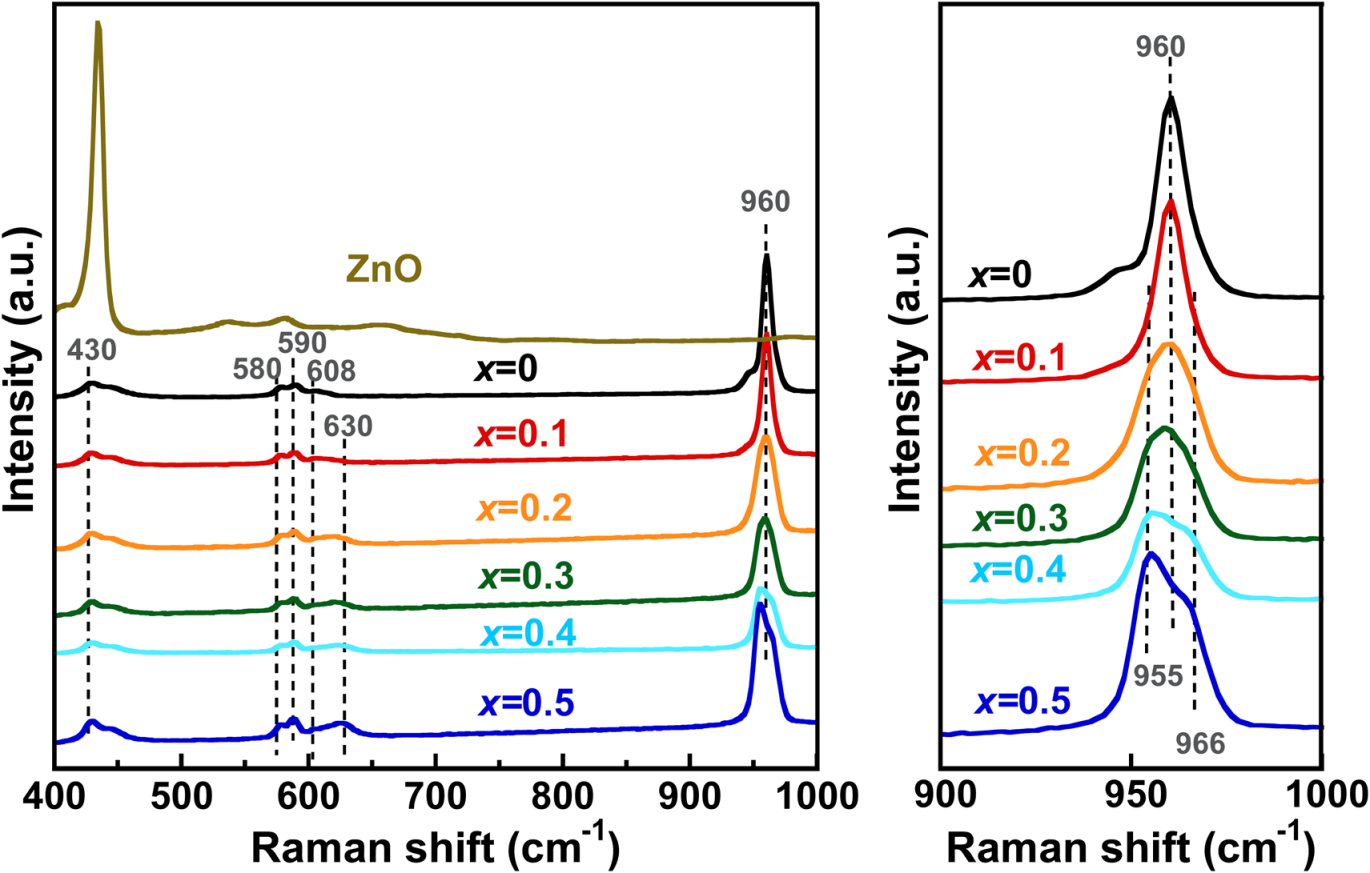
Raman spectra of Zn_*x*_-Hap together with a reference ZnO. An expanded version of the spectra is shown to the right.

The Raman spectra of the Zn_*x*_-Hap together with a reference ZnO are shown in Fig. 7. In addition to the absorption bands at 430, 580, 590, 608 and 960 cm^−1^, which are attributable to the PO_4_^3−^ of Hap,^87,88^ a band at 630 cm^−1^ is observed in the case of Zn-incorporated Hap, and the intensity increased with increases in the Zn content. In the case of [O–Cu–O] or [O–Ni–O] formation in the channel, absorption band appeared at 652 cm^−1^ or 690 cm^−1^, respectively.^50^ According to the inverse relationship between atomic mass and vibrational energy described by the spring model, bands ascribable to the metal–oxygen band are expected to be located at lower wavenumbers with heavier metals. Accordingly, the fact that the new band associated with Zn incorporation appears at a lower wavenumber (630 cm^−1^) compared to heavier metals (652 and 690 cm^−1^ for Cu and Ni, respectively) indicates that this new band can be attributed to [O–Zn–O] units. It should be noted that the Raman spectrum of ZnO does not exhibit any absorption band at this wavenumber. It is also suggested that the band at 960 cm^−1^ attributable to the phosphate group became broader with increases in Zn content up to *x* = 0.2, and that additional bands at 955 and 966 cm^−1^ appeared for *x* ≥ 0.3. These spectral changes are attributable to local distortion of the PO_4_^3−^ tetrahedra, which is caused by the incorporated Zn^2+^. This result correlates well with a previous report on Hap whose channel was modified by Zn.^70,76,77^

### 3.2 Zn deposition from hydroxyapatite

**Fig. 8:**
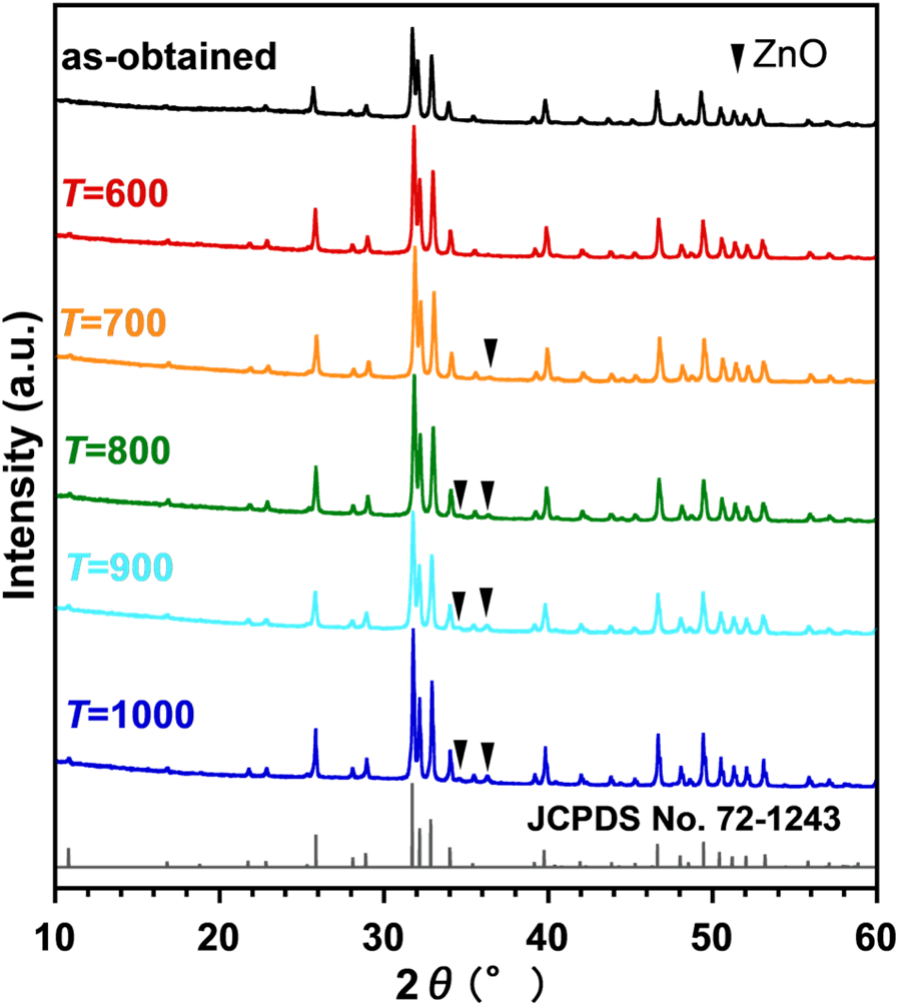
XRD patterns of products obtained by annealing Zn_0.4_-Hap at various temperatures for 3 h.

**Fig. 9:**
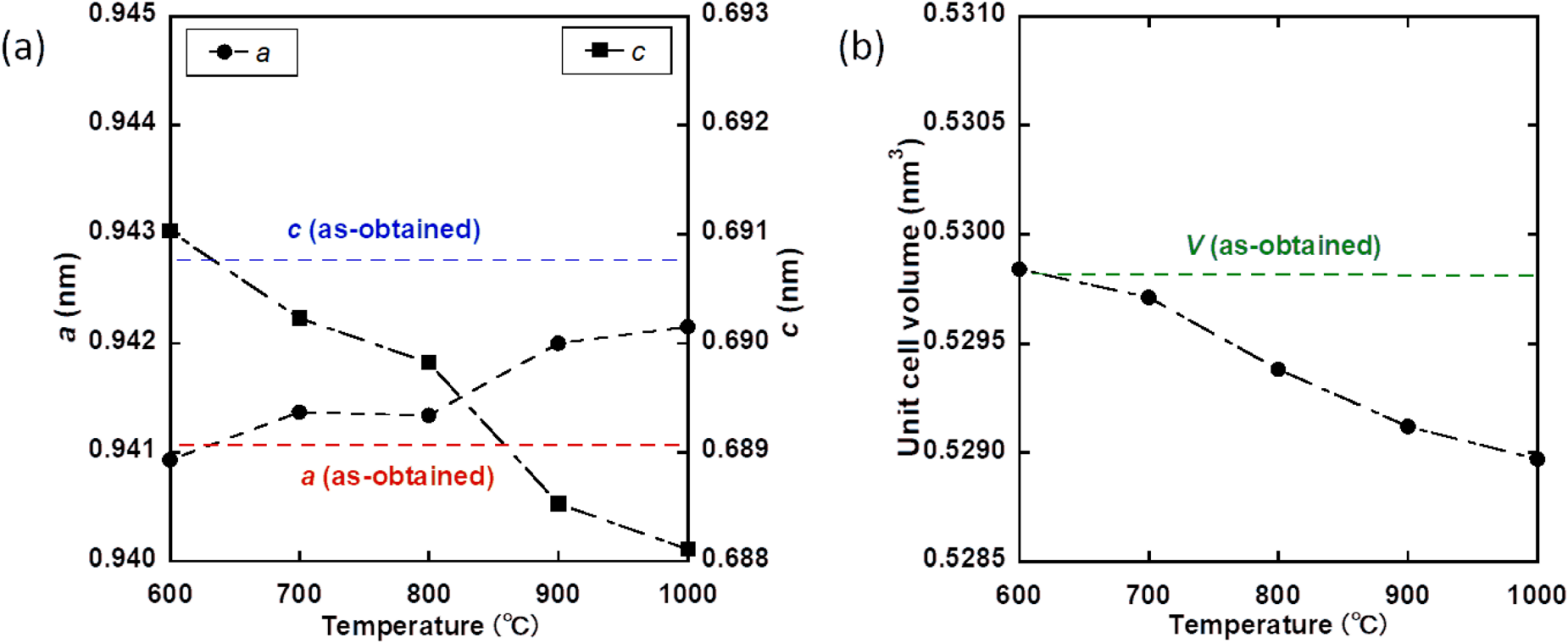
Lattice parameters (a) and unit cell volumes (b) of Zn_0.4_-Hap as a function of the annealing temperature.

To examine the deposition of Zn from the channel, Zn_0.4_-Hap was annealed in air at various temperatures for 3 h. The XRD patterns of the products are shown in Fig. 8. Except at 600 °C, the diffraction lines of 2*θ* = 36.4 with/without that of 34.4°, which are ascribable to ZnO, were observed for all the annealing temperatures, suggesting successful deposition of Zn as an oxide from the channel with the aid of H_2_O in accordance with eqn (1). The higher the annealing temperature employed, the larger the *a* and the smaller the *c* observed (Fig. 9a and Table S3). Accordingly, a smaller unit cell volume was observed as a higher annealing temperature was applied (Fig. 9b and Table S3). It should be noted that a negligible change in the lattice parameters was observed for pure Hap annealed at every temperature (Fig. S2, S3 and Table S3). Considering that opposite trends in the lattice parameters and unit cell volume were observed in the case of Zn incorporation into the channel, these results suggest that larger amounts of Zn were deposited from the channel with increases in the annealing temperatures in the range of ≥ 700 °C. On the other hand, the unit cell volume of Zn_0.4_-Hap annealed at 1000 °C (0.52897 nm^3^), which showed the smallest unit cell volume among the annealed products, was still larger than that of pure Hap treated in the same manner (0.52869 nm^3^), indicating that a portion of the Zn ions was still stabilized in the channel (Table S3). The deposition of larger amounts of Zn from the channel as higher annealing temperatures were employed fairly matches the fact that a higher intensity of the absorption band ascribable to OH^−^ at 632 cm^−1^ was observed with increases in the annealing temperature (Fig. S4), given that the proton uptake advances at the expense of Zn deposition, as discussed above. Diffusion pass of Zn during deposition process is of interest. Oxide-ion conduction,^89–91^ together with anion exchange reaction,^92^ is an example of atomic diffusion in apatite structure that has been continuously investigated. In the case of apatite-type lanthanum silicate, the path of oxide-ion migration parallel and perpendicular to the *c*-axis (channel) has been reported.^89,90^ On the other hand, path of cation migration in the deposition process that we have reported so far^47,50,51^ is unclarified. Given that the 1D channel is surrounded by Ca cations, migration of the metal in the channel as oxoanion, rather than as bare cation, is realistic in terms of electrostatic repulsion. In this case, diffusion perpendicular to the channel is unrealistic from the perspective of steric hindrance. Therefore, migration in the channel is a possible deposition path of Zn, while the mechanism is still controversial. From the perspective of the fineness of deposited ZnO, the product obtained at 700 °C, which was the lowest annealing temperature to achieve Zn deposition among the candidates, will be our focus hereafter. Assuming a correlation between Zn content *x* and the unit cell volume, the *x* of Zn_0.4_-Hap after annealing at 700 °C was estimated to be 0.34 based on its unit cell volume of 0.52971 nm^3^, given that the unit cell volume of pure Hap and as-obtained Zn_0.4_-Hap were 0.52906 and 0.52983 nm^3^, respectively.

**Fig. 10:**
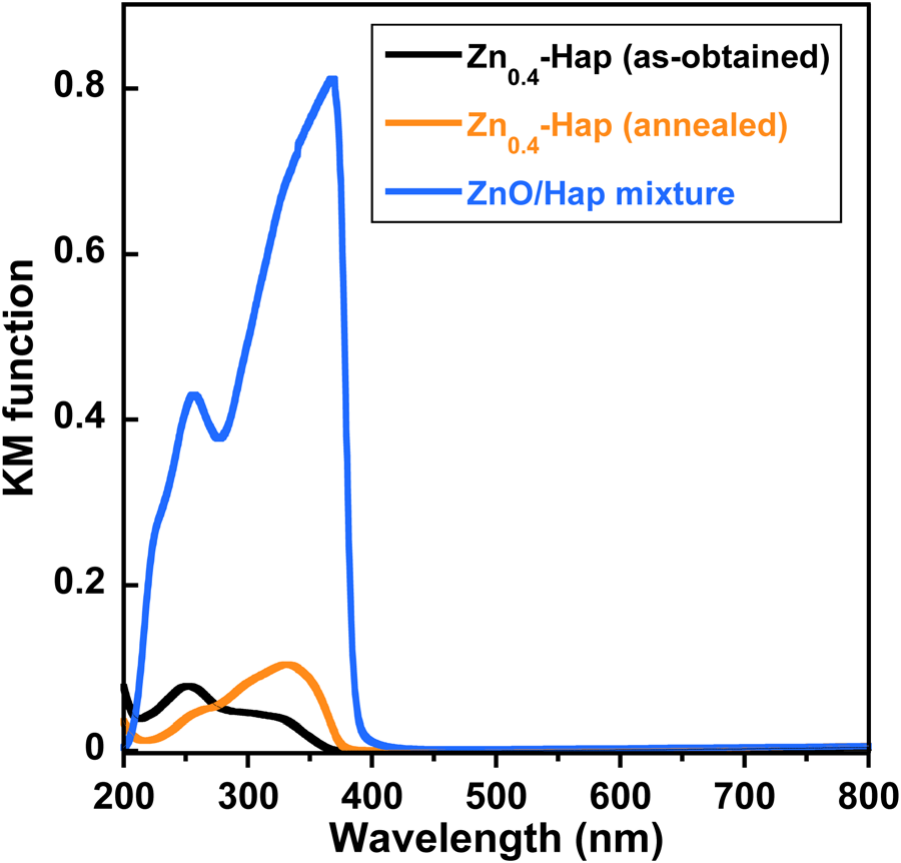
UV-vis DR spectra of Zn_0.4_-Hap before and after annealing at 700 °C, and the ZnO/Hap mixture. The ZnO/Hap mixing ratio was fixed assuming that all the Zn was deposited from Zn_0.4_-Hap.

The UV-vis DR spectra of Zn_0.4_-Hap before and after annealing at 700 °C for 3 h and the ZnO/Hap mixture are shown in Fig. 10. Light absorption in the wavelength range of 270–380 nm was increased upon annealing, while that in the wavelength range shorter than 270 nm was decreased. In the case of a physical mixture of pure Hap and a commercially available ZnO, the light absorption edge was located at 400 nm, equivalent to a band gap energy of ZnO (3.2 eV).^93^ The fact that the light absorption edge of Zn_0.4_-Hap after annealing is similar to the physical ZnO/Hap mixture indicates that the increased light absorption upon annealing is attributable to the deposited ZnO.

To examine the characteristics of ZnO particles depending on the synthetic route, ZnO was formed on Hap by the common impregnation method as a comparison of deposition from the channel. XRD patterns of products obtained by annealing the Hap wetted with an aqueous Zn(NO_3_)_2_·6H_2_O solution (the Zn/Ca molar ratio was set to 10 : 0.4) at *T* °C (*T* = 600–1000) for 3 h in air are shown in Fig. S5. All the XRD patterns show diffraction lines attributable to ZnO in addition to those ascribable to Hap, indicating successful conversion of Zn(NO_3_)_2_·6H_2_O into ZnO on Hap. Despite the formation of ZnO, no increase in the unit cell volume of Hap was observed at annealing temperatures of 600 or 700 °C, indicating that all the loaded Zn was converted into ZnO without incorporation into the channel. In the case of ≥ 800 °C, on the other hand, the unit cell volume increased with increases in the annealing temperature (Fig. S6 and Table S4). Moreover, the absorption band at 632 cm^−1^ attributable to OH^−^ in the IR spectra decreased, and absorption bands attributable to Zn^2+^ in the channel at 730 and 795 cm^−1^ were also observed at 900 and 1000 °C, suggesting incorporation of Zn into the channel (Fig. S7). The Hap whose surface was modified by ZnO directly at 700 °C is referred to as ZnO@Hap hereafter.

**Fig. 11:**
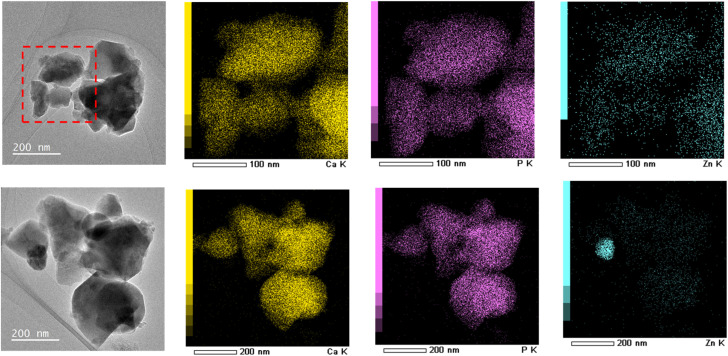
TEM images and the corresponding elemental mappings of Zn_0.4_-Hap (annealed) (upper) and ZnO@Hap (lower).

TEM images and the corresponding elemental mapping of Zn_0.4_-Hap after annealing and ZnO@Hap, both of which were obtained by annealing at 700 °C for 3 h, are shown in Fig. 11. While homogeneous distribution of the Zn element was observed in the TEM elemental mapping of annealed Zn_0.4_-Hap (Fig. 11 upper), the concentration of Zn was confirmed in the case of ZnO@Hap (Fig. 11 lower). Given that the Ca/Zn molar ratios of both samples (0.034 and 0.041 for annealed Zn_0.4_-Hap and ZnO@Hap, respectively, as shown in Table S5) were almost identical to the nominal composition (0.040), these results indicate more homogeneous distribution of ZnO particles on annealed Zn_0.4_-Hap when compared to ZnO@Hap.

**Fig. 12:**
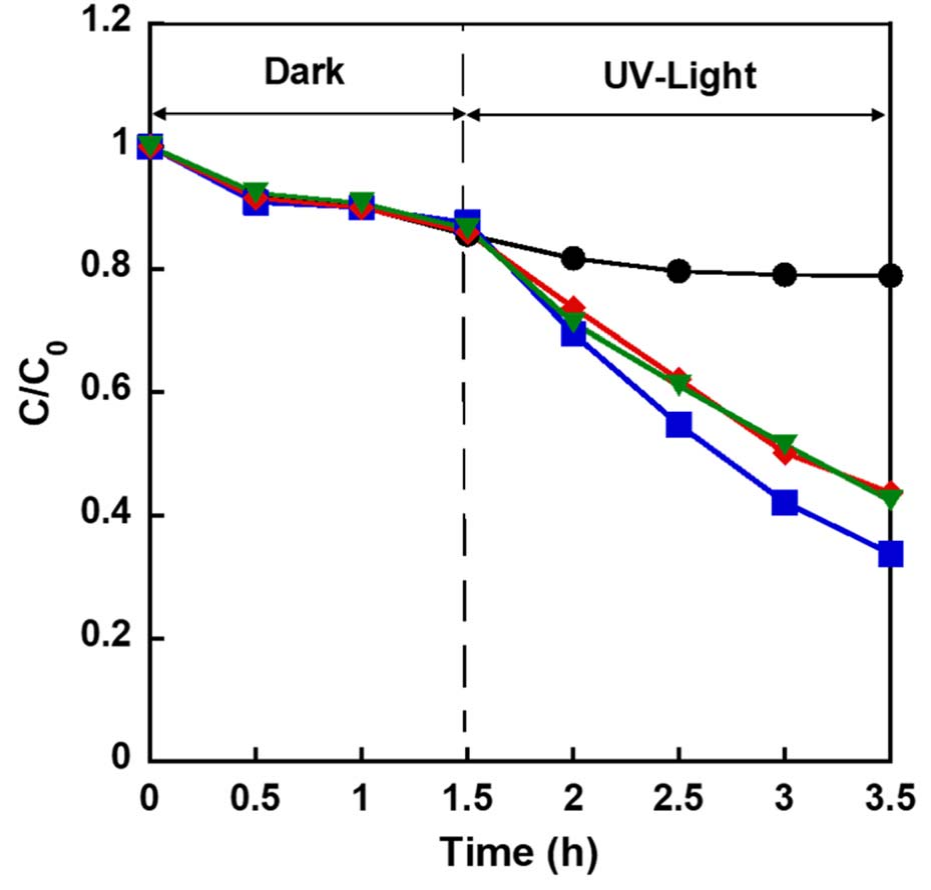
Changes in the *C*/*C*_0_ of MB in the presence of Zn_0.4_-Hap (as-obtained) (black), Zn_0.4_-Hap annealed for 3 h (blue) or 9 h (red) and ZnO@Hap (green) under dark and light irradiation.

Fig. 12 shows the change in the *C*/*C*_0_ of aqueous MB solution in the presence of different samples under dark/light irradiation. In the case of as-obtained Zn_0.4_-Hap, *C*/*C*_0_ was almost constant over extended periods of time, indicating that the photocatalytic performance of inert Hap for MB decomposition^94^ was not modified by Zn incorporation into the channel. On the other hand, *C*/*C*_0_ decreased in the cases of both annealed Zn_0.4_-Hap and ZnO@Hap under light irradiation, indicating that ZnO photocatalyst immobilized on these products decomposed MB. To correlate surface area (fineness) of ZnO deposited from the channel and annealing time period, decomposition rate of MB at Zn_0.4_-Hap annealed for 9 h was compared with the case of 3 h. The *C*/*C*_0_ after light irradiation for 2 h was 0.337 and 0.437 for the annealing time period of 3 and 9 h, respectively. The shorter the annealing time period applied, the smaller the *C*/*C*_0_ observed, indicating decreased photocatalytic activity by extending the annealing time period to more than 3 h. Considering that the unit cell volume of annealed Zn_0.4_-Hap was almost identical for both cases (Table S6), it is unlikely that the amount of deposited ZnO was increased by extending the annealing time period. The decrease in photocatalytic activity upon further annealing thus indicated decrease in surface area (particle growth) of deposited ZnO. The fact that a negligible diffraction line ascribable to ZnO became remarkable by extending the span of annealing time from 3 h to 9 h (Fig. S8) supports this scenario. It should be noted here that intensity of the diffraction lines of Hap in annealed Zn_0.4_-Hap unchanged upon light irradiation in aqueous MB solution (Fig. S8), suggesting stability of host apatite lattice over the examined span of irradiation time. To investigate fineness of ZnO particle on Hap depending on immobilizing methodology, Zn_0.4_-Hap annealed for 3 h was compared with ZnO@Hap in terms of MB decomposition efficiency. The *C*/*C*_0_ after light irradiation for 2 h in the case of annealed Zn_0.4_-Hap (0.337) was lower than that for ZnO@Hap (0.424), suggesting superior photocatalytic activity of annealed Zn_0.4_-Hap when compared with ZnO@Hap. The content of ZnO immobilized on the annealed Zn_0.4_-Hap is smaller (15.6%) compared to that of ZnO@Hap, considering that the *x* of annealed Zn_0.4_-Hap was 0.34 as discussed above based on the unit cell volume, while it was suggested that all the loaded Zn was converted into ZnO in ZnO@Hap. Given that the annealed Zn_0.4_-Hap showed superior photocatalytic activity compared to ZnO@Hap despite its remarkably lower ZnO content, a higher specific surface area of ZnO deposited from the channel, when compared to ZnO directly deposited on the Hap surface by the conventional impregnation method was indicated, a result correlating with the ZnO distribution suggested by TEM electron mapping. These results demonstrate the usefulness of the incorporation–deposition process of guest metal ions into the channel of Hap as a means of addressing Hap decorated by fine particles of metal oxides, which is applicable to catalyst design.

## Conclusions

Loading of Zn onto hydroxyapatite and subsequent annealing under a dry atmosphere significantly enhanced the efficiency of Zn incorporation into the channel of hydroxyapatite up to a maximum incorporation level of 5 mol% with respect to Ca while maintaining a single-phase structure, surpassing the limit achieved by the conventional wet synthesis method. The site occupied by Zn was clarified to be the 2*b* site. Annealing treatment in air was employed to investigate the behavior of Zn in Zn-containing hydroxyapatite, revealing that Zn ions incorporated into the channel tend to be deposited as ZnO on the hydroxyapatite surface. Based on the evaluation of the ZnO distribution on hydroxyapatite together with the photocatalytic activity of the ZnO-hydroxyapatite composites thus obtained, incorporation and deposition of Zn^2+^ into the channel of hydroxyapatite was suggested to be a promising means of immobilizing ZnO fine particles on the hydroxyapatite surface. The insights gained in the present study suggest the usefulness of the post synthetic route for design of hydroxyapatite-based functional hybrids applicable to catalysts.

## Author contributions

Xiao Chen: investigation, writing – original draft, writing – review and editing. Kanji Saito: conceptualization, investigation, writing – original draft, writing – review and editing. Takashi Toyao: investigation, writing – original draft, writing – review and editing. Yuriko Ando: investigation, writing – review and editing. Ken-ichi Shimizu: supervision, writing – review and editing. Masataka Ogasawara: supervision, writing – review and editing. Sumio Kato: supervision, writing – review and editing.

## Conflicts of interest

There are no conflicts of interest to declare.

## Supplementary Material

RA-015-D5RA06364G-s001

## Data Availability

All data supporting the findings are available within the article. Supplementary information is available. See DOI: https://doi.org/10.1039/d5ra06364g.
