# Supplementary material for: Incorporation and deposition behaviors of Zn into the channel of hydroxyapatite
Source: RSC Adv. 2025 Oct 14;15(45):38147–57. doi: 10.1039/d5ra06364g (PMC12519319; doi:10.1039/d5ra06364g)
Supplement: RA-015-D5RA06364G-s001 [file RA-015-D5RA06364G-s001.pdf]

Supplementary Information (SI) for RSC Advances.
This journal is © The Royal Society of Chemistry 2025

Supporting Information

Incorporation and deposition behaviors of Zn into the channel of hydroxyapatite

Xiao Chen[a], Kanji Saito[a,b*], Takashi Toyao[c], Yuriko Ando[c], Ken-ichi Shimizu[c], Masataka Ogasawara[a] and Sumio Kato[a*]

[a] Department of Materials Science, Graduate School of Engineering Science, Akita University, 1-1 Tegatagakuen-machi, Akita-shi, Akita 010-8502, Japan

[b] Kagami Memorial Research Institute for Materials Science and Technology, Waseda University, 2-8-26 Nishiwaseda, Shinjuku-ku, Tokyo 169-0054, Japan

[c] Institute for Catalysis, Hokkaido University, N-21, W-10, Sapporo 001-0021, Japan

Table of Contents

Fig. S1 Lattice parameters (a) and unit cell volumes (b) of Zn_x-Hap as functions of *x*. 3

Table S1 Lattice parameters and unit cell volumes of the Zn_x-Hap 3

Table S2 Refined structural parameters of $Zn_{0.4}$-Hap assuming that the Zn occupies the 12*i* site............. 3

Fig. S2 XRD patterns of Hap annealed at various temperatures. 4

Fig. S3 Lattice parameters (a) and unit cell volumes (b) of Hap as a function of the annealing temperature. 4

Table S3 Lattice parameters and unit cell volumes of $Zn_{0.4}$-Hap and Hap annealed at various temperatures 5

Fig. S4 IR spectra of $Zn_{0.4}$-Hap with/without annealing at various temperatures. 5

Table S4 Lattice parameters and unit cell volumes of products obtained by annealing the Hap wetted with an aqueous $Zn(NO_3)_2 \cdot 6H_2O$ solution (the Zn/Ca molar ratio was set to 10 : 0.4) at *T* ℃ (*T* = 600-1000) in air 5

Fig. S5 XRD patterns of products obtained by annealing Hap wetted with an aqueous $Zn(NO_3)_2 \cdot 6H_2O$ solution (the Zn/Ca molar ratio was set to 10 : 0.4) at *T* ℃ (*T* = 600-1000) in air. 6

Fig. S6 Lattice parameters (a) and unit cell volumes (b) of products obtained by the Hap wetted with an aqueous $Zn(NO_3)_2 \cdot 6H_2O$ solution (the Zn/Ca molar ratio was set to 10 : 0.4) as a function of annealing temperature. 6

Fig. S7 IR spectra of products obtained by annealing Hap wetted with an aqueous $Zn(NO_3)_2 \cdot 6H_2O$ solution (the Zn/Ca molar ratio was set to 10 : 0.4) at *T* ℃ (*T* = 600-1000) in air. 7

Table S5 The Zn/Ca molar ratio of each sample obtained from EDS measurement.................................. 7

Table S6 Lattice parameters and unit cell volumes of $Zn_{0.4}$-Hap annealed at 700 ℃ for 3 or 9 h 7

.... 8

Fig. S8 XRD patterns of $Zn_{0.4}$-Hap annealed for 3 h (black) and 9 h (red), and the product obtained by annealing $Zn_{0.4}$-Hap for 9 h after light irradiation in aqueous MB solution (blue). 8

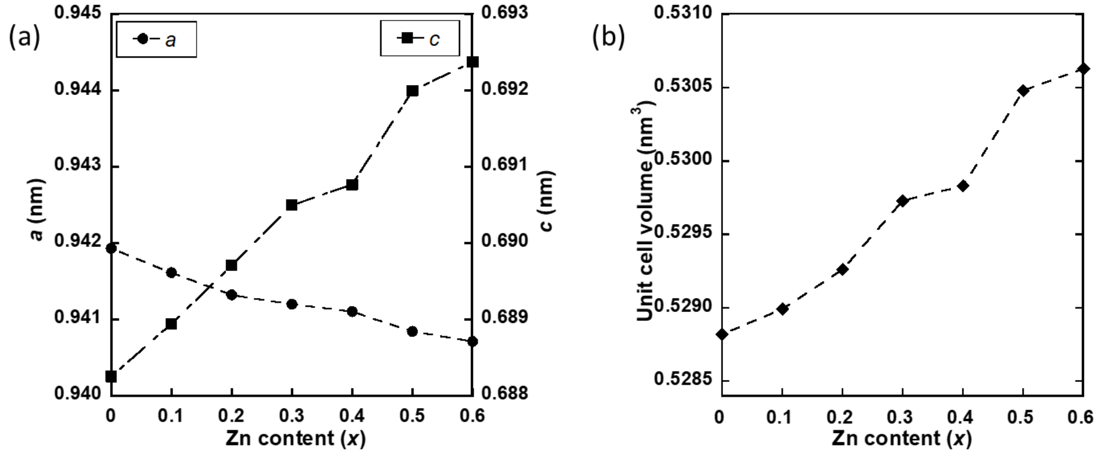

Fig. S1 Lattice parameters (a) and unit cell volumes (b) of Zn_x-Hap as functions of *x.*

Table S1 Lattice parameters and unit cell volumes of the Zn_x-Hap

Atmosphere	*T* (°C)	*x*	*a* (nm)	*c* (nm)	V(nm^3)
Air (wet)	1150	0	0.941746(14)	0.688269(12)	0.528636(15)
		0.1	0.94161(7)	0.68879(5)	0.52889(7)
		0.2	0.94158(4)	0.68918(3)	0.52915(4)
		0.3	0.94151(9)	0.68948(7)	0.52930(9)
		0.4	0.94103(10)	0.69045(7)	0.52951(10)
		0.5	0.94093(10)	0.69101(7)	0.52982(10)
N_2	900	0	0.94193(4)	0.68825(3)	0.52882(4)
		0.1	0.94161(10)	0.68894(8)	0.52899(10)
		0.2	0.94132(4)	0.68971(3)	0.52926(4)
		0.3	0.94120(8)	0.69050(6)	0.52973(8)
		0.4	0.94110(10)	0.69077(7)	0.52983(9)
		0.5	0.94084(10)	0.69200(8)	0.53048(11)
		0.6	0.94071(7)	0.69238(5)	0.53063(7)

Table S2 Refined structural parameters of $Zn_{0.4}$-Hap assuming that the Zn occupies the 12*i* site

Space group; $P6_3/m$ (NO.176); *a*=0.94072(12) nm, *c*=0.69109(6) nm, V=0.52965(10) nm^3, R_{wp}=3.729%, R_F=1.175%

Atom	Site	*g*	n	*x*	*y*	*z*	U($100nm^2$)
O1	6h	1	6	0.3284(3)	0.4841(3)	1/4	0.0139(8)
O2	6h	1	6	0.5866(3)	0.4640(3)	1/4	0.0127(8)
O3	12i	1	12	0.3413(2)	0.2559(2)	0.0695(2)	0.0185(6)
O4	4e	0.5	2	0	0	0.2153(15)	0.021(2)
P	6h	1	6	0.3987(1)	0.3685(2)	1/4	0.0132(4)
Ca1	4f	1	4	1/3	2/3	0.0017(2)	0.0141(4)
Ca2	6h	1	6	0.2462(1)	-0.0075(1)	1/4	0.0149(3)
Zn	12i	0.0334(5)	0.4005(56)	0.023(2)	0	0	0.020(4)

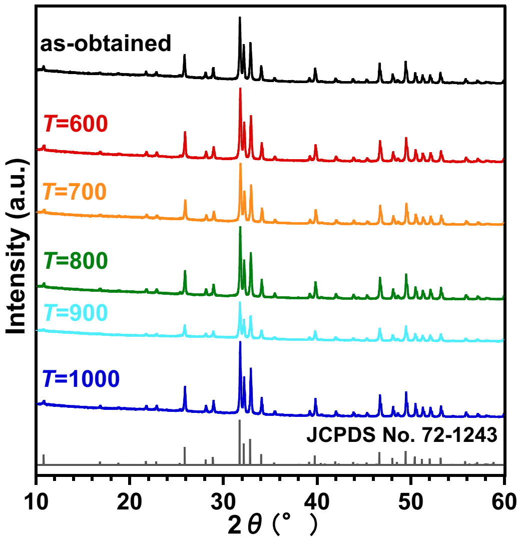

Fig. S2 XRD patterns of Hap annealed at various temperatures.

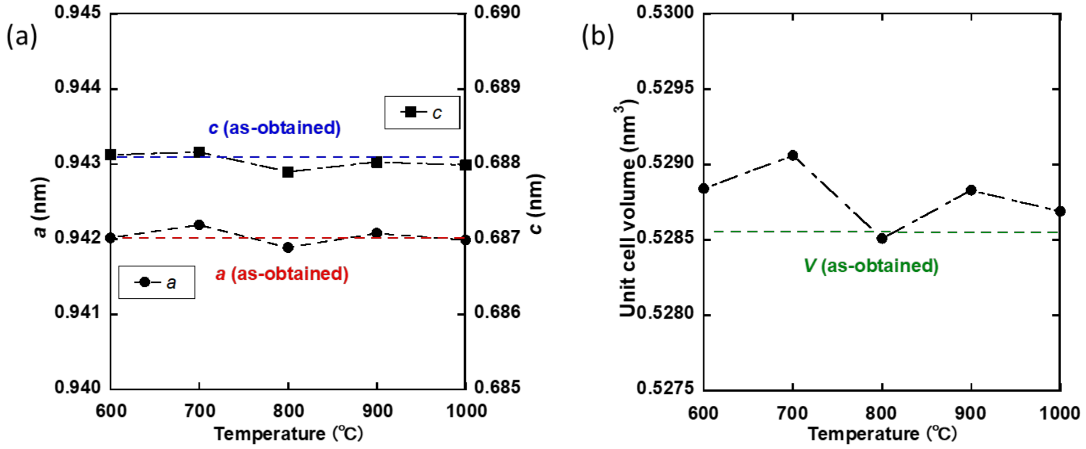

Fig. S3 Lattice parameters (a) and unit cell volumes (b) of Hap as a function of the annealing temperature.

Table S3 Lattice parameters and unit cell volumes of $Zn_{0.4}$-Hap and Hap annealed at various temperatures

sample		*a* (nm)	*c* (nm)	V(nm^3)
Hap annealed	As-obtained	0.94179(3)	0.68809(2)	0.52856(3)
	600	0.94202(8)	0.68813(6)	0.52884(7)
	700	0.94219(3)	0.68816(2)	0.52906(3)
	800	0.94189(6)	0.68790(4)	0.52851(6)
	900	0.94208(4)	0.68803(3)	0.52883(4)
	1000	0.94199(6)	0.68799(5)	0.52869(6)
$Zn_{0.4}$-Hap annealed	As-obtained	0.94110(10)	0.69077(7)	0.52983(9)
	600	0.94093(10)	0.69103(7)	0.52984(10)
	700	0.94137(13)	0.69023(9)	0.52971(12)
	800	0.94134(10)	0.68982(8)	0.52938(10)
	900	0.94200(7)	0.68853(5)	0.52912(7)
	1000	0.94215(5)	0.68811(4)	0.52897(5)

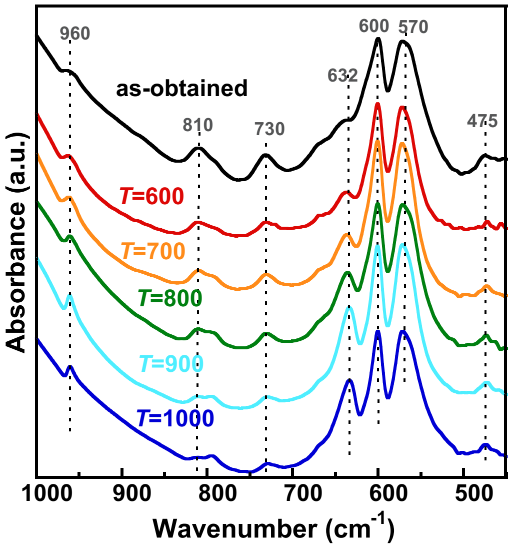

Fig. S4 IR spectra of $Zn_{0.4}$-Hap with/without annealing at various temperatures.

Table S4 Lattice parameters and unit cell volumes of products obtained by annealing the Hap wetted with an aqueous $Zn(NO_3)_2 \cdot 6H_2O$ solution (the Zn/Ca molar ratio was set to 10 : 0.4) at *T* °C (*T* = 600-1000) in air

T (°C)	*a* (nm)	*c* (nm)	V(nm^3)
600	0.94123(10)	0.68824(7)	0.52803(9)
700	0.94134(7)	0.68807(5)	0.52802(6)
800	0.94153(6)	0.68796(5)	0.52816(6)
900	0.94183(7)	0.68839(5)	0.52883(7)
1000	0.94184(6)	0.68877(4)	0.52912(6)

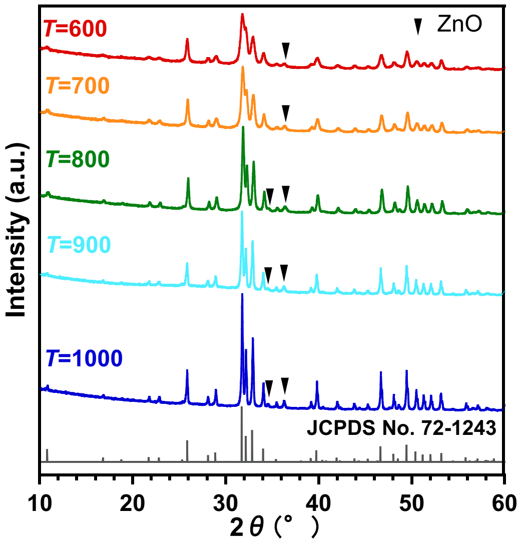

Fig. S5 XRD patterns of products obtained by annealing Hap wetted with an aqueous $Zn(NO_3)_2 \cdot 6H_2O$ solution (the Zn/Ca molar ratio was set to 10 : 0.4) at *T* °C (*T* = 600-1000) in air.

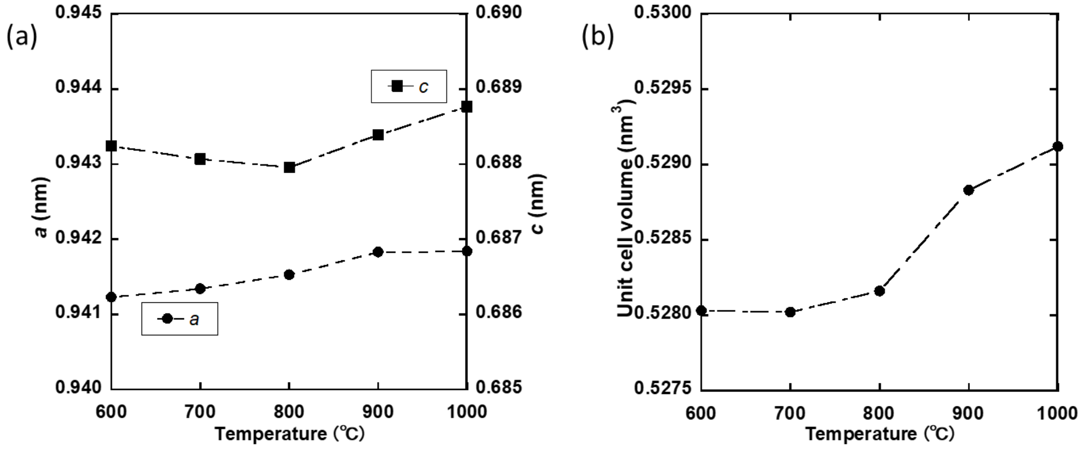

Fig. S6 Lattice parameters (a) and unit cell volumes (b) of products obtained by the Hap wetted with an aqueous $Zn(NO_3)_2 \cdot 6H_2O$ solution (the Zn/Ca molar ratio was set to 10 : 0.4) as a function of annealing temperature.

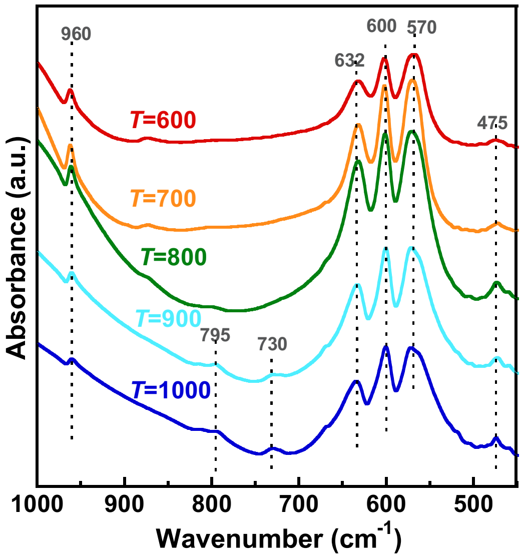

Fig. S7 IR spectra of products obtained by annealing Hap wetted with an aqueous $Zn(NO_3)_2 \cdot 6H_2O$ solution (the Zn/Ca molar ratio was set to 10 : 0.4) at *T* °C (*T* = 600-1000) in air.

Table S5 The Zn/Ca molar ratio of each sample obtained from EDS measurement

Sample	Element	wt %	mol %	Zn/Ca molar ratio
$Zn_{0.4}$-Hap annealed	Ca	36.18	21.1	0.034
	P	17.14	12.93	
	O	44.67	65.25	
	Zn	2.02	0.72	
ZnO@Hap	Ca	36.41	21.08	0.041
	P	15.11	11.32	
	O	46.02	66.73	
	Zn	2.46	0.87	

Table S6 Lattice parameters and unit cell volumes of $Zn_{0.4}$-Hap annealed at 700 °C for various time periods

sample	*a* (nm)	*c* (nm)	V(nm^3)
3h	0.94136(9)	0.69065(7)	0.53002(9)
9h	0.94149(8)	0.69050(6)	0.53005 (8)

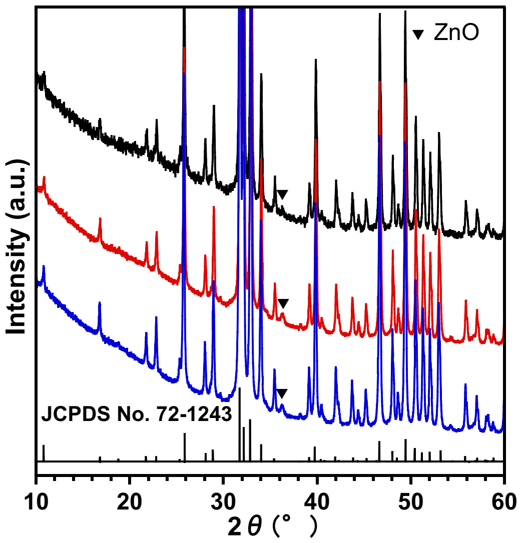

Fig. S8 XRD patterns of $Zn_{0.4}$-Hap annealed for 3 h (black) and 9 h (red), and $Zn_{0.4}$-Hap obtained by annealing for 9 h and subsequent light irradiation in aqueous MB solution (blue).
